# Normalized stability time analysis within the boundaries between adults with and without fear of falling

**DOI:** 10.1007/s40520-023-02651-0

**Published:** 2024-01-28

**Authors:** Dongchul Lee, Paul S. Sung

**Affiliations:** 1 Nevro Inc., 1800 Bridge Parkway, Redwood City, CA USA; 2 Doctor of Physical Therapy Program, Indiana Wesleyan University, 4201 South Washington Street, Marion, IN 46953 USA

**Keywords:** Fear of falling, Normalized stability time, Unilateral standing, Thresholds, Visual input

## Abstract

**Background:**

The unilateral stance test, measured by the center of pressure (COP), has been widely used to identify balance deficits. However, there is a critical gap in understanding the specific COP thresholds on postural stability in adults with a fear of falling (FOF).

**Aims:**

To investigate the normalized stability time, which was defined as the ratio of time spent within stability boundaries to the total test duration, under different visual conditions and specific thresholds between adults with and without FOF.

**Methods:**

Twenty-one older adults with FOF and 22 control subjects completed the unilateral limb standing test in eyes-open and eyes-closed conditions. Normalized stability times were computed based on five pre-determined COP sway range thresholds: 10 mm, 15 mm, 20 mm, 25 mm, and 30 mm.

**Results:**

Receiver operating characteristic analysis determined the diagnostic accuracy of FOF. There were significant differences in the effects of both visual conditions (*F* = 46.88, *p* = 0.001) and threshold settings (*F* = 119.38, *p* = 0.001) on stability time between groups. The FOF group significantly reduced normalized stability time at the 10 mm COP threshold under eyes-closed conditions (*t* = – 1.95, *p* = 0.03).

**Discussion:**

The findings highlight the heightened sensitivity of the 10 mm COP threshold in identifying group variances in postural stability when eyes are closed. Moreover, the FOF group displayed a marked reduction in stability duration based on visual scenarios and normalized thresholds.

**Conclusion:**

The study highlights the need to account for both COP boundaries and visual conditions in adults with FOF. When assessing postural control during unilateral stances, clinicians must also give attention to non-visual cues.

## Introduction

Falls in older adults are one of the most common causes of a variety of physical impairments that affect gait, balance, and functional activities [1]. Many individuals also experience fear of falling (FOF), which is defined as a sense of concern regarding the dangers of falling that is sufficient to impede one's participation in daily activities [2]. This phobic reaction to standing or walking is associated with serious physical and psychosocial consequences for balance confidence, which may result in functional decline and death [3–6]. Several studies reported that physical activity in individuals with visual impairments can lead to an important risk factor for fall injuries [7–9]. However, these studies lacked a consensus to adopt a standard way to analyze accurate dynamic postural stabilities and related falls while considering visual input.

The fall risks reflect compensatory reactions due to common tasks of daily living, such as unilateral stance during walking. Clinicians utilize the unilateral standing test as one of the most valuable balance tests because it is widely considered to be cost-effective and feasible in clinical research settings [10, 11]. Previous studies reported postural stability during unilateral standing to assess time-to-boundary (TTB) of postural control [12–14]. Their approaches quantify postural stability in unilateral stance for the assessment of TTB of postural control. Although the TTB measures were comparable to traditional COP, the correlations between TTB and traditional measures based on spatial (range) or temporal (velocity) components of COP excursion were much less consistent due to different aspects of postural control in single limb stance than traditional variables [15, 16]. However, their assessment and traditional measures were weak or not sensitive to detect changes based on the sway ranges [17].

In our study, a time-in-boundary (TIB) based on normalized stability time within specific thresholds was analyzed to detect postural stability during unilateral standing. A TIB analysis is a measurement to compute the total time which subjects keep the COP within the 'hypothetical circle' during unilateral standing. These hypothetical circles were used as various levels of threshold (10 mm, 15 mm, 20 mm, 25 mm, and 30 mm) to determine stability. A normalized relative stability time percent analysis in individuals with and without FOF may provide additional clinical insights. Although balance problems are the most common reasons for falls, it is important to provide a tool to detect sensitive changes based on the COP sway ranges. Therefore, it is important to provide a tool to detect sensitive changes based on the COP sway ranges within the COP boundaries. Evidently, a recent study supported our study in terms of setting boundaries to objectively analyze results for better accuracy in detecting postural deficits [18].

There is a lack of understanding on the postural stability analysis within various boundaries of thresholds when considering visual input as well as other counfounding factors. These factors are related to musculoskeletal problems, which might influence the course of central nervous system compensation and balance recovery [19]. For example, visual input has been critical to compensate during unilateral standing [20–22], especially in individuals with FOF. Other individual characteristics, such as body mass index (BMI), gender, and the role of limb dominance, are critical in unilateral standing tests [23]. Without controlling for these confounding factors, the results of balance analysis could lead to limited generalizability of the outcome measures, which produces incorrect clinical interpretations.

We theorize that normalized relative stable times within the COP boundaries might provide a propensity to maintain a postural correction, especially in individuals with FOF. Clinicians may need information on postural reactions with visual input following unilateral stance in order to develop rehabilitaion strategies for individuals with FOF. Therefore, the purpose of this study was to investigate (1) groups with and without FOF based on the cut-off value to infer a new reference value by the receiver operating characteristic (ROC) analysis, and (2) TIB within various threshold boundaries during unilateral limb standing between individuals with and without FOF. It was hypothesized that the FOF group would demonstrate reduced TIB from the COP in the eyes-closed condition during unilateral limb standing compared to the control group. We expected that individuals in the FOF group would exhibit different values of reduced TIB from the COP during eyes-closed, unilateral limb standing as compared to the control group. We hypothesized this difference based on prior research indicating that FOF significantly impacts postural control mechanisms, especially when visual input is removed [20–22].

## Methods

Subjects were recruited from the community through advertisements. Eligible individuals were between the ages of 50 and 75 years, right-limb dominant, and had no history of limb pain for at least three months prior to the study. They were also free from any serious pathology, such as nerve root compromise. Exclusion criteria included a diagnosed psychological illness, overt neurological signs, or pregnancy. Age and BMI were considered in recruiting the control group.

During the consent process, each subject was given standardized procedures to measure the outcomes of the test during unilateral standing with and without visual input. Subjects were asked whether they had a history of falls and if they had to interrupt certain activities (by responding with binary yes/no answers). A summary of the test and measure tools in our study is shown in Table 1. The FOF refers to the apprehension about the potential risks of falling that is strong enough to limit an individual’s engagement in daily activities [24]. This concept is particularly relevant to older adults who may be concerned about experiencing a fall, whether or not they have fallen before. Subjects completed a 10-item Falls Efficacy Scale (FES) questionnaire to classify them into FOF and non-FOF groups. Other clinical measures used for assessment included the Oswestry Disability Index (ODI) and the Timed Up and Go (TUG) test. Subjects were also required to perform unilateral standing tasks under various visual conditions as part of the screening process.

**Table 1:** Description of the test/measure tools for the study

Test/measure	Purpose	Description
Falls Efficacy Scale (FES)	The FES was utilized to quantify the degree of perceived efficacy in daily activities [24]	The questionnaire consists of 10 items, which have 10 possible quantified responses ranging from 1 to 10. A dichotomous classification as FOF group (yes = 1; no = 0) was used in the analysis based on the median score of the FES
Oswestry Disability Index (ODI)	The level of disability was measured by the ODI, which consists of 10 items regarding the degree of severity to which back (or limb) trouble has affected the ability to manage activities in everyday life [25]	The 10 sections cover pain and daily function, and each item is rated on a 6-point scale; the higher score means a higher level of disability
Timed Up and Go (TUG) test	The TUG test is a tool to identify a patient’s functional mobility and risk of falling, especially older adults [26]	The TUG test is a reliable, cost-effective, safe, and time-efficient way to evaluate overall functional mobility. The TUG has a high correlation with other proven tests that measure pure gait speed for longer lengths, such as a 10-m walk
Unilateral standing test	The relative stability time tolerance was measured within the thresholds during unilateral standing [21, 27]	The test protocol utilized the Bertec Balance Advantage^®^ system, a computer-controlled, motorized platform capable of Computerized Dynamic Posturography with Immersion Virtual Reality

Upon arrival, demographic data were collected. Subjects were asked to stand on the Bertec Balance Advantage^®^ system, a computer-controlled, motorized platform capable of Computerized Dynamic Posturography with Immersion Virtual Reality (CDP-IVR). Ground Reaction Forces (GRF) were captured using a force plate (Bertec, Columbus, Ohio) with a sampling frequency of 1000 Hz. All kinetic data were filtered and normalized based on individual body weight. Upon arrival at the lab, individual demographic data were collected.

Subjects were instructed to remove their footwear and to stand barefoot on the platform. Subjects wore a full-body safety harness system that imposed negligible resistance and protected them from any potential injuries if they completely lost their balance; however, the harness did not affect the subjects’ balance recovery or assist them in any way. The tension on the safety straps was adjusted, so the straps were neither too slack nor too taught. The experimental protocol included subjects standing on the computer-controlled, motorized Bertec Balance Advantage^®^ system with their feet placed at a comfortable distance apart.

The medial malleolus of each foot of each subject was positioned over the blue horizontal line on the support surface, so that the ankle joint was aligned with the transverse rotational axis and the lateral side of the calcaneous. The y-axis indicated AP movements on the platform, while side-to-side movements on the support surface occurred along the x-axis. The dual force plates can rotate about the x-axis, which represents the transverse axis of the ankle joint. This position acts as a reference point for the calculation of sway angles.

Each subject was instructed to remain on his/her dominant foot during the trial based on visual condition. Subjects were asked to stand barefoot on one limb for 10 s, while flexing the contralateral knee at approximately 30° behind them and maintaining a vertical limb position to the standing limb. For example, a subject was asked to stand steady on the dominant foot for 10 s with his/her eyes open (or closed) on the balance plate. The initial position included standing relaxed with the eyes open. Though the subjects began each trial with their arms at their sides, compensatory arm movements were permitted to maintain balance.

The force plate (Bertec, Columbus, Ohio) was used to record the GRF (Fx, Fy, and Fz) in orthogonal directions at a sampling frequency of 1000 Hz. The manufacturer calibrated the force plate, and a sensitivity matrix was provided to convert the voltages to forces and torques. The data was collected from the unloaded platform to determine the zero offset, and the balance changes imposed during one-legged stance balance tasks were utilized. Force plate data represented a combination of both disturbance and postural control reaction when subjects were engaged in a balance task typically employed to measure postural sway. The force plates are the ‘gold standard’ for balance testing, and plates have been shown to exhibit moderate to very high reliability across a range of postural sway measures [28]. All kinetic data were filtered using a fourth-order low-pass Butterworth filter with a 20 Hz cut-off frequency, and normalization was performed based on individual body weight.

The COP sway path lengths (mm) were analyzed based on the linear measures root mean square (RMS) and range (max–min) for the AP and ML directions [29, 30]. These parameters were independent of the effect of body weight, and those linear measures quantify the amount of variability in the data. Therefore, the COP refers to the point of application of the GRF vector, and it describes the organization of posture.

**Fig. 1:**
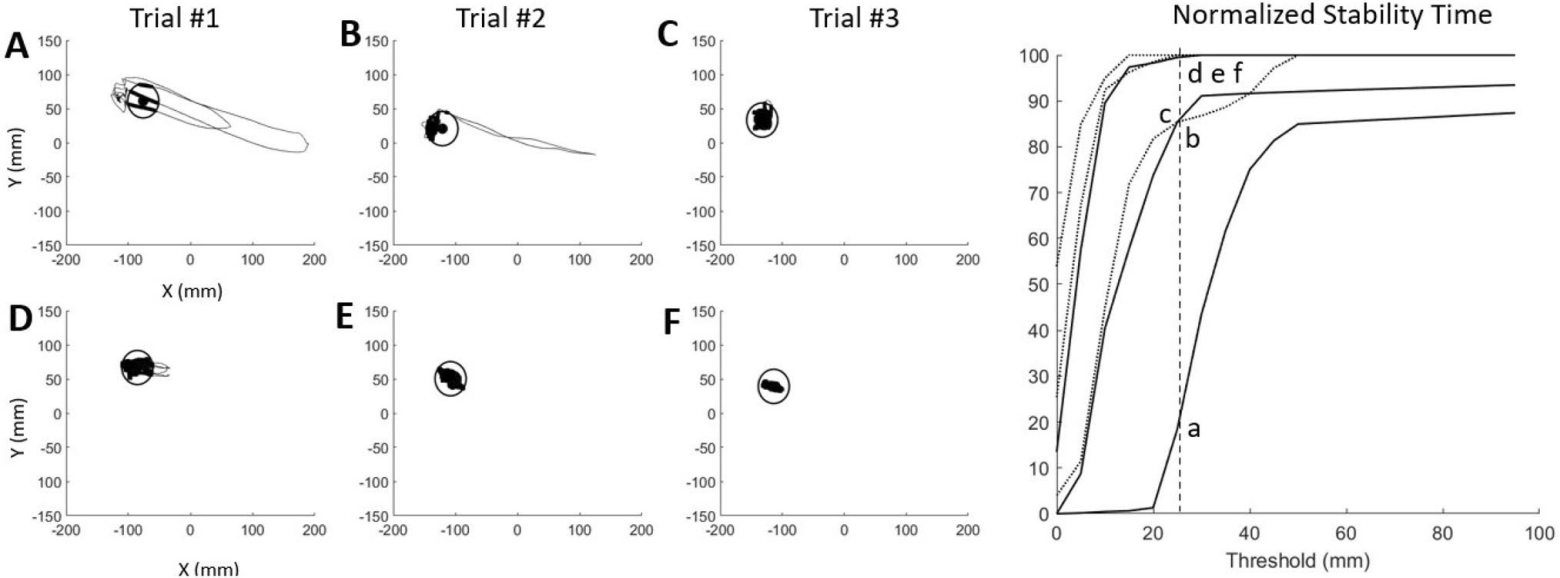
An example of the threshold circle (radius = 25 mm), which was drawn from the trajectory center for 10 s. A normalized stability time with COP was calculated during dominant limb standing on a platform in the eyes-open condition. **A** First trial of the trajectory was plotted with the medio-lateral location as the x-axis and the anteroposterior location as the y-axis for a subject with FOF. Failing the unilateral standing task shows deviation from the center point (black dot) of the trajectory. The data points within the threshold circle were plotted as dark lines. **B** and **C** Second and third trials of the same subject. The trajectory of COP from the third trial stayed mostly within the threshold circle. **D**–**F** Standing trial with a healthy subject. The first trial shows a moment out of the threshold circle; however, the second and third trials were all within the threshold circle. **G** The normalized stability time percent was the relative time of trajectory staying within a threshold circle during 10 s standing, which was calculated as a function of the threshold. The normalized relative stable time increased as the threshold got bigger. The vertical dotted line is the example threshold (25 mm) used in **A** through **F**. The lower-case alphabet letters (a–f) correspond to each **A**–**F**. For example, subject **A** maintained standing posture for 18% of relative stability; however, subject **C** was able to maintain 85% of relative stability within the 25 mm threshold in standing. The normalized stability time decreased in a subject with fear of falling during the trial

The TIB used only COP (x, y), which is a final outcome of posture control in a standing stability test. The TIB counts only the data points within the threshold (distance from the mean value of COP during the test) without penalizing the sporadic sway during posture correction. If there are multiple sways moving away during the test, the mean of the COP will be affected by those sways. A TIB analysis is a measurement to compute the total time in which subjects keep the COP within the ‘hypothetical circle’ during unilateral standing. These hypothetical circles were used as various levels of threshold (10 mm, 15 mm, 20 mm, 25 mm, and 30 mm) to determine stability. To calculate the relative stability in each threshold boundary measure, the AP and ML directions of the COP were used. Figure 1 indicates an example of the threshold circle (radius = 25 mm), which was drawn from the trajectory center between a subject with and without FOF during three repeated trials of the dominant limb standing task.

Statistical analyses were completed using SPSS 26.0 (IBM Corp, Armonk NY, USA). ROC analysis was used to determine the sample-specific cut-off point for prediction of FOF that minimized the total number of misclassification errors. A dichotomous classification as FOF group (yes = 1; no = 0) was used in the ROC analysis. Preliminary power analyses were conducted based on the pilot data comparing groups. The effect sizes were confirmed by partial eta-squared values (*η*^2^*p*) within repeated measures ANOVA squared (small ≥ 0.01, medium ≥ 0.06, large ≥ 0.14), which was used to indicate the mean difference between groups. The independent variables included groups (with and without FOF). The mixed repeated measure analysis of variance (ANOVA) was utilized between groups to analyze any main and/or interaction effects on the degree of kinesiophobia and fall efficacy. The demographic factors, such as age and BMI, as well as ODI and TUG, were used as covariates if a group difference was revealed. For all statistical tests, the type I error rate was set at 0.05.

## Results

As shown in Table 2, the study included 21 subjects with FOF (made up of 13 females and 8 males) and 22 control subjects (made up of 15 females and 7 males). Statistical analyses revealed no significant differences between groups in terms of gender distribution (*χ*^2^ = 0.66, *p* = 0.75), age (*t* = – 0.14, *p* = 0.86), or BMI (*t* = – 0.26, *p* = 0.79). However, the FOF group exhibited significantly higher scores for the ODI (*t* = – 2.69, *p* = 0.01) and TUG test (*t* = – 3.03, *p* = 0.004) compared to the control group. These variables were used as covariates in subsequent analyses. In addition, no significant group differences were observed regarding previous history of falls within the past year (*χ*^2^ = 0.73, *p* = 0.39).

**Table 2:** Summary of subject anthropometric variables and measurements between groups

Variables	Control group	Fear of falling group	Statistics	*p*
Number of subjects (Female/Male)	22 (15/7)	21 (13/8)	*χ*^2^ = 0.66	0.75
Age (years)	63.27 ± 7.42	63.67 ± 10.29	*t* = – 0.14	0.86
BMI (kg/m^2^)	23.60 ± 5.98	24.04 ± 4.52	*t* = – 0.26	0.79
ODI	8.82 ± 11.11	21.20 ± 18.11	*t* = – 2.69	0.01*
TUG	8.59 ± 1.34	9.99 ± 1.68	*t* = – 3.03	0.004**
FES	0.91 ± 1.30	0.98 ± 0.05	*t* = 2.09	0.02*
Fall (Yes/No)	3/19	5/16	*χ*^2^ = 0.73	0.39

*BMI* body mass index, *ODI* Oswestry Disability Index, *TUG* Timed Up and Go, *FES* Fall Efficacy Scale, *Fall* history of falls in the last year, (Average ± standard deviation)

*p < 0.05

**p < 0.01

**Fig. 2:**
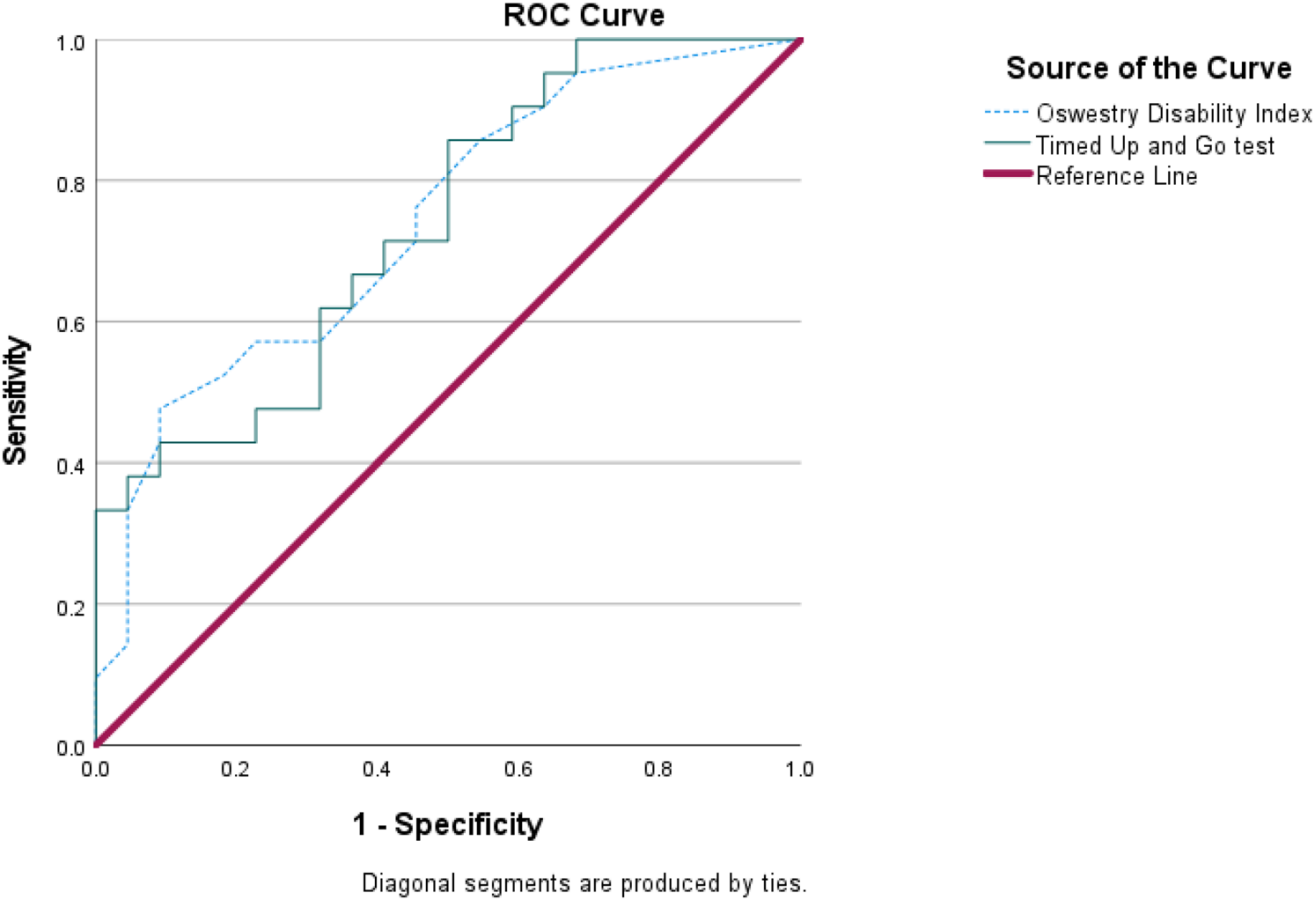
ROC curves of cut-off values for predicting fall risks with the ODI and TUG test. AUC results for cut-off values with ODI of fall risks are 0.74 (*p* = 0.01; confidence interval lower limit: 0.59 and upper limit: 0.89), and cut-off values with TUG of fall risks are 0.73 (*p* = 0.01; confidence interval lower limit: 0.59 and upper limit: 0.88). The ROC curves identified older adults with fall risks. The ROC curve reveals the probability of an individual with a trait (e.g., fall history) to be correctly identified. The greater the discrimination performance, the closer the AUC is to 1. If the AUC is less than 0.5, the probability of identifying older adults with and without fall efficacy is random. *ROC* receiver operating characteristics, *AUC* area under the curve

Figure 1 data points plotted within the threshold circle revealed that the normalized relative stability time increased in subjects without FOF under the same conditions. All subjects successfully completed the unilateral standing test for the requested duration throughout the test protocol. The kinetic data showed that the COP sway path lengths in both the ML and AP directions were assessed. An example of the results indicated that the trajectory of the COP stayed mostly within the threshold circle during a unilateral standing task in older adults without FOF. As shown in Fig. 2, the AUC results suggest that both tests are reliable indicators for assessing the likelihood of falling among the subjects. Therefore, these findings support the utility of ODI and TUG as valuable tools in fall risk assessments.

**Fig. 3:**
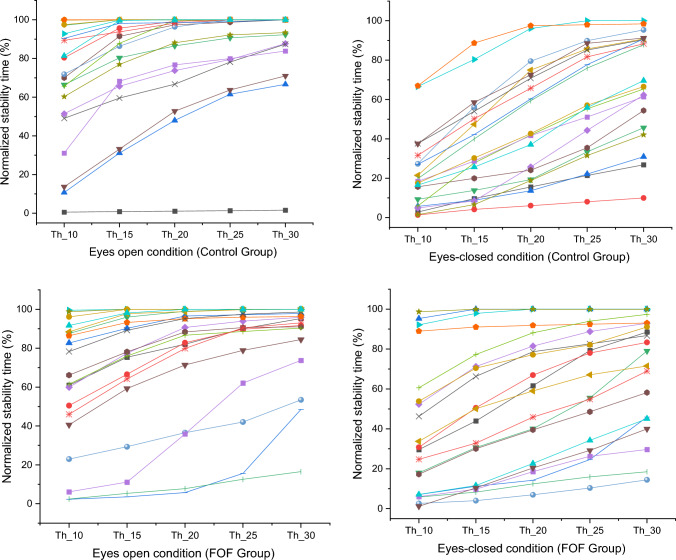
Plots with separate lines were distributed for each subject during unilateral standing trials. The trends of normalized stability time response (time-in-boundary) on visual condition were similar within the group. In the eyes-open condition, the control group demonstrated increased normalized stability time compared with the fear of falling (FOF) group. During the eyes-closed condition, however, the normalized stability time increased in the FOF group at 10 mm thresholds

**Fig. 4:**
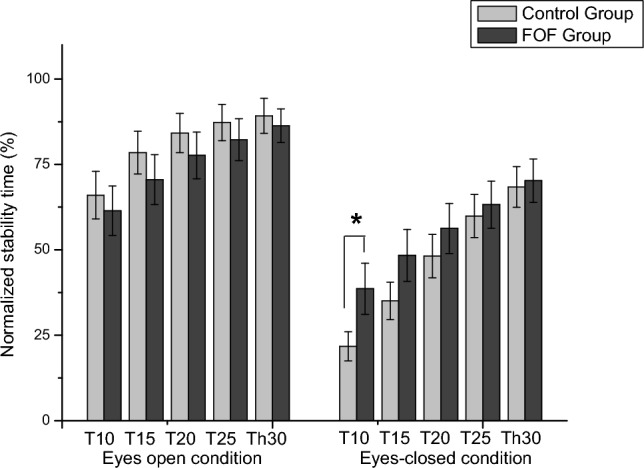
The relative stability time tolerance within the thresholds during unilateral standing. The sway ranges were analyzed for various thresholds (10 mm, 15 mm, 20 mm, 25 mm, and 30 mm) from the center of pressure with and without visual input. There was a significant group interaction between visual conditions and thresholds (*F* = 6.03, *p* = 0.02). The control group demonstrated significantly decreased stability time at the 10 mm threshold in the eyes-closed condition (*t* = – 1.95, *p* = 0.03). *T* combined thresholds of the anteroposterior and medio-lateral boundaries, *FOF* fear of falling, *p < 0.05

Figure 3 displays varied stability time responses between groups under different visual conditions. Specifically, those without FOF had increased stability time under the eyes-open condition. There were significant variations in stability times between groups, particularly under different visual conditions and COP thresholds. The normalized relative stability time within the threshold boundaries was analyzed by the mixed repeated measure ANOVA (Table 3). The results indicated that the groups demonstrated a significant interaction on the visual conditions and thresholds (*F* = 6.03, *p* = 0.02, *η*^2^*p* = 0.14). There were significant differences on the visual conditions (*F* = 46.88, *p* = 0.001, *η*^2^*p* = 0.55) and thresholds (*F* = 119.38, *p* = 0.001, *η*^2^*p* = 0.84) as well as interactions between visual conditions and thresholds (*F* = 17.79, *p* = 0.001, *η*^2^*p* = 0.32). More importantly, as shown in Fig. 4, the groups demonstrated a significantly different TIB on the 10 mm threshold in the eyes-closed condition (38.60 ± 7.47 in the FOF vs. 21.76 ± 4.27 in the control group; *t* = – 1.95, *p* = 0.03).

**Table 3:** Results of mixed repeated measure ANOVA for the unilateral standing trials between groups

Variables	*F*	*p*	Partial Eta Squared (*η*^2^*p*)
Visual condition	46.88	0.001**	0.55
Visual condition × group	3.10	0.08	0.07
Thresholds	119.38	0.001**	0.84
Thresholds × group	3.32	0.14	0.06
Visual condition × thresholds	17.79	0.001**	0.32
Visual condition × thresholds × group	6.03	0.02*	0.14

*p < 0.05

**p < 0.01

## Discussion

Our study had two primary objectives. The first was to determine a new reference value for FOF based on the cut-off value of the measurement tools. Our results suggest that using the area under the ROC curve provides both construct and predictive validity for classifying older adults into groups with and without FOF. The area under the ROC curve was sensitive to FOF among participants, which reinforces its discriminative capacity. The second objective was to compare the TIB based on the normalized stability time during unilateral limb standing between individuals with and without FOF. We observed significant differences in normalized stability time based on visual conditions and thresholds. Our approach, focusing on normalized relative stability, provided better detection capabilities for dynamic balance control, particularly in various threshold levels for sway excursions.

The FOF groups demonstrated significantly increased TIB on the 10 mm threshold in the eyes-closed condition to protect them from falls. There were significant normalized stability time differences on the visual conditions and thresholds. Previous studies reported limited information based on TTB measures, which captured postural compensations during quiet standing to detect postural deficits [12, 18]. In addition, a meta-analysis report indicated poor sensitivity of the COP spatial-based measures due to the overlooked temporal aspect of balance [31]. Their results imply that TTB measures assess different aspects of postural control in single limb stance than traditional variables.

Our data analyses build upon the limitations of previous studies that used TTB measures, which yielded inconsistent results and failed to consider the temporal aspect of balance [17]. Our study addresses these gaps by incorporating normalized stability time as a sensitive metric for detecting balance deficits during unilateral limb standing. The TIB counts only the data points within the threshold (distance from the mean value of COP during the test), without penalizing the sporadic sway during posture correction. The normalized relative stability analysis provides better detection of dynamic balance based on the various levels of threshold for sway excursions. The unilateral standing test is one of the most used balance tests and is widely considered to be cost-effective and feasible in clinical research settings [10, 11].

Several systematic reviews validated the clinical importance of unilateral standing balance measures [32–35]. If there are multiple sways moving away during the unilateral test, the mean of the COP will be affected by sway ranges in AP and ML directions. This pattern will eventually lower the TIB values. If the COP excursion is random, it may or may not affect the TIB. If the COP variation is large, it will lower the TIB because any COP points out of threshold will not be counted as stable. Our TIB analysis reflects available sensory information (such as proprioception [unilateral stance] and vision [visual constraint]), which has helped to explain how subjects with balance deficits related to their clinical problems manage to cope with somatosensory absences based on the specific thresholds of TIB. The TIB metric enhances the existing body of research by incorporating available sensory information and specific threshold levels, which appear to be more sensitive in identifying balance deficits.

Our research emphasizes the significance of sensory input in regulating postural control. Specifically, vision was highlighted as vital for sustaining balance, especially in the eyes-closed condition. In this condition, the balance proficiency of subjects with FOF became evident during a 10-s unilateral stance. To maintain equilibrium, these subjects increased their dependence on vision as an alternate system when others were compromised. The threshold for the TIB concerning COP limits was distinctly effective in distinguishing groups at a 10 mm excursion threshold during eyes-closed sessions. Given the varying visual conditions, the different TIB thresholds proved to be credible metrics for assessing unilateral limb standing in individuals with FOF.

We hypothesized that the FOF group would demonstrate reduced TIB from the COP in the eyes-closed condition during unilateral limb standing compared to the control group. We partially accepted this hypothesis since the FOF group demonstrated significantly decreased normalized stability time at the 10 mm threshold in the COP boundaries during the eyes-closed condition. The visual conditions and thresholds to the TIB were critical to the relative stable time within the boundaries between groups. In other studies, successive COP measurements were evident when considering visual conditions [36, 37], and the significance of the visual effect was supported by our results in compensatory mechanisms of balance in the eyes-open condition. In addition, somatosensory dependency characteristics of postural control may compromise balance in older adults to compensate for errors and to stabilize the system [38, 39]. The sensory information used for postural control mainly arises from the vestibular system of the inner ear, vision, and proprioception [40, 41]. These sources of sensory information for postural control might be different in older adults with FOF.

In our study, subjects with FOF potentially exhibited increased postural sway, typically surpassing the minimum detectable change. This observation suggests that identifying heightened postural sway is an efficient method to address fall risks across various TIB thresholds. Our mixed repeated measure ANOVA results showed significant interactions between the groups based on visual conditions and TIB threshold boundaries concerning normalized relative stability times. There were significant differences in visual conditions, TIB thresholds, and their interactions. Furthermore, the FOF group registered a decrease in normalized stability time within the TIB during eyes-open scenarios. Our subjects with FOF displayed patterns of asymmetrical weight-bearing and a forward-leaning posture.

As a result, the stochastic activity and positively correlated (persistent) behavior of the postural sway during shorter timescales may cause postural instability [42]. The results of other studies suggest a close integration of biomechanical and goal-related constraints in perception and control of body orientation. Although balance performance for unilateral standing required a reduction in postural stability, the results of our analyses based on the TIB were sensitive to differentiate only at the 10 mm boundary in the eyes-closed condition. This threshold provided the amount of time tolerance available to make corrective postural adjustments, which would allow clinicians to track the magnitudes of the threshold boundaries to make these adjustments. Our results for the TIB in the eyes-open condition did not demonstrate a significant difference and was not critical for balance assessment. A visual-related rehabilitation strategy should at least involve mobility-related movement component(s) or form part of a multi-component training to achieve a beneficial effect on balance.

A recent meta-analysis summarized that balance performance is not influenced by limb dominance as the performances of both limbs can be used as a reference [23]. Their results were based on healthy adults, but visual condition in adults needs to be carefully considered with other individual factors based on age- and BMI-matched samples. Our study focused exclusively on the standardized unilateral balance test in right-limb dominant subjects in addition to similar characteristics of older adults during unilateral standing. The balance test and fall risks reflect compensatory reactions because most common tasks of daily living involve standing on one limb, which removes the ability to compensate. Another review indicated that the influence of limb dominance on postural balance would be context-dependent as one single factor may not be enough to impact postural balance [43].

Our study underscores the value of the unilateral stance test when factoring in visual input. The assessment of postural sway ranges within a hypothetical circle offers a promising tool for rehabilitation evaluation. For subjects with FOF, rehabilitating without relying on visual input becomes pivotal for postural control to further functional recovery. Our findings offer insights into identifying sensitive measures of COP sway ranges across various thresholds. If subjects can maintain their balance within a given threshold, even with minor disturbances, the TIB measurement is deemed stable. Conversely, a widely scattered COP without a discernible steady state point could negatively impact the TIB value. Individuals with compromised balance often display reduced body sway capacity. Understanding this can aid in refining intervention strategies targeting COP sway boundaries. Such impaired balance might be tied to musculoskeletal functionality and heightened muscle tension [44]. It is anticipated that this conceptual expansion of the theoretical model of stability to one with the symbiotic inclusion of mobility may provide new understandings on human movement [45].

There were several limitations in our study. The demographic variations might invalidate the results even if age- and BMI-matched older adults participated in the study. The subgroup analyses would provide more accurate results of intra/inter-variability for future studies. In addition, the subjects’ characteristics were not restricted based on postural deficits or fall episodes. Although one examiner gave the standardized instructions during the test, possible individual variations on the unilateral test and related motion artifacts may have affected individual scores. Further studies are warranted to improve postural equilibrium strategies to help dynamic balance and control while considering visual input in older adults with FOF.

The clinical relevance of our study lies in its potential balance strategies for those with FOF. Our findings can guide clinicians in assessing postural control and balance deficits, thereby facilitating functional recovery. The TIB measure provides a robust tool for rehabilitation assessments, especially when considering the importance of visual input. Future work should aim to validate these findings in more diverse populations and to further explore the impact of visual condition on balance.

## Conclusion

The FOF group demonstrated significantly increased normalized stability time at the 10 mm threshold of COP boundaries in the eyes-closed condition. Our findings imply that the adults with FOF need to enhance their fall-related confidence during unilateral standing tasks. The visual conditions and thresholds to the TIB were critical to differentiate normalized stability time between groups.

## Data Availability

The data that support the findings of this study are not openly available due to the conditions of ethics approval for the study and current data protection legislation. Dependent on compliance with data protection legislation and ethical approval, they may be available from the corresponding author upon reasonable request.
